# A Narrative Review of Evidence, Safety, and Clinical Considerations in Taxane Chemotherapy for Pregnancy-Associated Breast Cancer

**DOI:** 10.3390/biomedicines13112635

**Published:** 2025-10-27

**Authors:** Jenny W. Zhang, Ochuwa Precious Imokhai, Danny Lee, Diana Hamdan, Trisha Mahajan, Satyam K. Singh, Amanda Brooks

**Affiliations:** 1 Montana College of Osteopathic Medicine, Rocky Vista University, Billings, MT 59106, USA; 2 Department of Medicine, University of California, San Francisco St. Mary’s Hospital, San Francisco, CA 94117, USA; 3 College of Osteopathic Medicine, Kansas City University, Joplin, MO 64804, USA; 4 Department of Medicine, Texas College of Osteopathic Medicine, Fort Worth, TX 76107, USA; 5 College of Medicine, University of Illinois, Chicago, IL 60612, USA; 6 Department of Research in Scholarly Activity, College of Osteopathic Medicine, Rocky Vista University, Ivins, UT 84738, USA

**Keywords:** pregnancy-associated breast cancer (PABC), fetal outcomes, maternal safety, taxanes, chemotherapy

## Abstract

The medical condition of pregnancy-associated breast cancer (PABC) requires oncologists to determine the best way to protect both the mother and the fetus during cancer treatment. The safety profile of taxanes, including paclitaxel and docetaxel, in the second and third trimesters of pregnancy remains unclear despite well-established anthracycline-based regimens (e.g., doxorubicin). High-risk breast cancer subtypes such as triple negative breast cancer (TNBC) and human epidermal growth factor receptor (HER2)-positive disease require taxane chemotherapy as standard treatment in nonpregnant patients. **Objective:** This paper aims to gather available data about the safety, timing and fetal outcomes related to taxane chemotherapy during PABC, focusing on pharmacological and clinical guidance. **Methods:** A targeted literature review of PubMed and Scopus databases was performed to identify case series, cohort studies, and clinical guidelines addressing taxane use during pregnancy. This was not conducted as a formal systematic review or meta-analysis, but as a comprehensive narrative synthesis of available data. **Results:** The pharmacological properties of paclitaxel and docetaxel limit their placental transfer. Paclitaxel has not been associated with increased congenital anomalies; however, the long-term developmental data remain limited. Similarly, docetaxel administration shows no increase in major malformations. The most common approach used in PABC is to administer anthracyclines first and taxanes after 16–18 weeks’ gestation. The adverse effects experienced by pregnant patients match those experienced by nonpregnant patients. **Conclusions**: Taxanes can be used with caution after the first trimester in patients with PABC, especially in high-risk cases following anthracycline treatment. The absence of randomized trials combined with limited developmental data highlight the need for more standardized treatment approaches, aligning with current guideline recommendations.

## 1. Introduction

Breast cancer is among one of the most prevalent malignancies associated with pregnancy [1]. Currently, pregnancy-associated breast cancer (PABC) is estimated to be around 1 in 3000 pregnancies with progressively higher risks with delayed childbearing [2,3]. This presents a unique challenge as physicians must balance optimal maternal treatment while minimizing the risks to fetus health. Among the chemotherapeutic agents, anthracyclines such as doxorubicin are considered relatively safe for administration during the second and third trimesters [4]. Taxanes, such as paclitaxel and docetaxel, are another chemotherapeutic agent commonly used to treat breast cancer, lung cancer, gastric cancer, and ovarian cancer. They are a critical chemotherapy agent in the management of both early and metastatic biologically aggressive breast cancer subtypes, including triple-negative breast cancer (TNBC) and HER2-positive disease [5]. However, the role of taxanes in PABC treatment remains limited due to ethical constraints that restrict clinical trials in pregnant populations. The objective of this review is to therefore examine current evidence on safety, efficacy, maternal, and fetal outcomes associated with taxane chemotherapy during pregnancy.

## 2. Method

**Figure 1:**
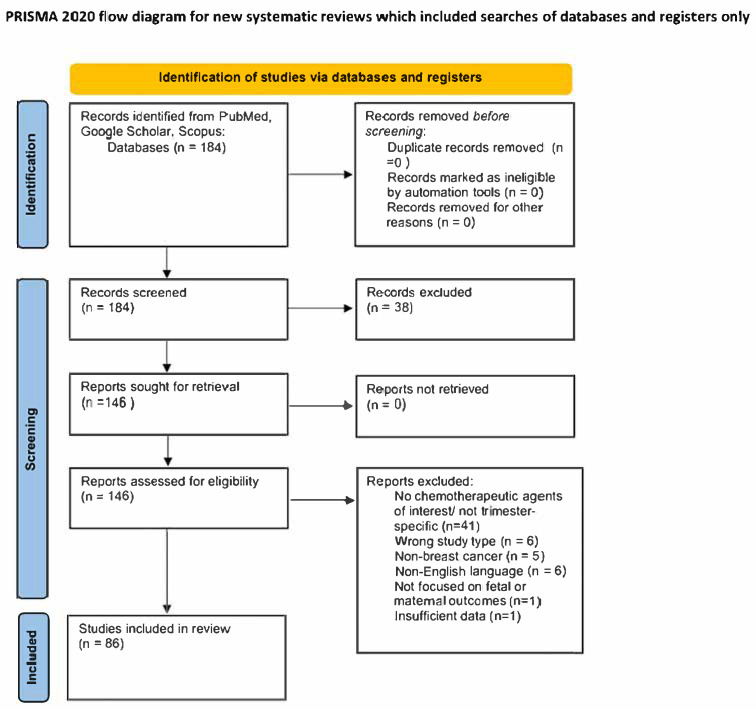
Literature screening and selection flow diagram (adapted from the PRISMA 2020 framework to illustrate transparency in article inclusion).

This narrative review aimed to summarize and critically appraise the current evidence on the safety of taxane chemotherapy during pregnancy. To enhance transparency, elements from systematic methodologies (e.g., structured database search, predefined inclusion/exclusion criteria, and PRISMA-style flow diagram) were incorporated. However, this study does not qualify as a systematic review or meta-analysis since risk-of-bias assessments, dual reviewer extraction, and quantitative synthesis were not conducted. The purpose was to provide a clinically oriented synthesis rather than an exhaustive systematic evaluation. We included clinical studies, case series, and relevant guidelines published in English that evaluated maternal and/or fetal outcomes following taxane exposure. We excluded articles unrelated to the topic (e.g., wrong cancer type, non-English language, and insufficient data). The search was not conducted or reported as a formal systematic review; rather, it was intended to provide a comprehensive overview of available literature. No meta-analysis was performed due to substantial heterogeneity in study design and reported outcomes, which limits the ability to draw pooled quantitative conclusions. A flow diagram (Figure 1) is provided to illustrate the literature selection process.

### 2.1. Search

In September 2025, a literature search using Pubmed and Scopus databases was conducted for studies related to taxane use in PABC between January 2000 and August 2025. Database search strategies included combining terms related to pregnancy, breast cancer, fetal outcomes, chemotherapy, and taxanes (including paclitaxel and docetaxel). Reference lists of eligible articles were also reviewed to include additional relevant studies.

### 2.2. Screening and Analysis

Eligible studies included cohort studies, case series, and clinical guidelines that evaluated maternal and/or fetal outcomes following exposure to taxane chemotherapy during pregnancy. A total of 184 records were identified from PubMed and Scopus. A total of 146 records were sought for retrieval and all 146 were assessed for eligibility. A total of 60 of these studies were excluded for the following reasons: No chemotherapeutic agents of interest/not trimester specific (*n* = 41), wrong study type (*n* = 6), non-breast cancer (*n* = 5), non-English language (*n* = 6), not focused on fetal or maternal outcomes (*n* = 1), and insufficient data (*n* = 1). A total of 86 reviews met all inclusion criteria and were analyzed as part of the final review. Full-text publications meeting inclusion criteria were retrieved and independently assessed by three reviewers, with any discrepancies being resolved through discussion and consultation with a fourth reviewer. The study inclusion process is summarized in Figure 1 and was carried out in adherence to the Preferred Reporting Items for Systematic Reviews and Meta-Analyses (PRISMA) guidelines. This review did not require institutional review board approval because it did not involve human participants or original data collection at any specific hospital or clinical site.

## 3. Discussion

### 3.1. Chemotherapy Management Trimester Based Approach

Most chemotherapy agents are rated pregnancy category D, in which there is evidence of human fetal risk, but potential benefits may be acceptable despite the risks [6]. Chemotherapy management in pregnant breast cancer patients should factor not only disease stage, but also maternal survival and fetal safety. Chemotherapy usually is not dose adjusted by pregnancy status, but dose-dense regimens have been proposed to allow for shorter periods of exposure [7,8]. Thus, a trimester-based approach is necessary. Risk to the fetus is highest during the first trimester (~20% of fetal malformation), when organogenesis occurs [9]. Chemotherapy is contraindicated during the first trimester due to the high risk of teratogenicity to the fetus.

Starting from the second trimester, chemotherapy is generally administered safely. Chemotherapy pharmacokinetics change during pregnancy, partly due to placental drug transfer, fetal organ maturity, maternal clearance, and volume distribution. Anthracyclines including doxorubicin, are efficacious to treat breast cancer and have a long history of safety during pregnancy [10]. Combination regimens are commonly used. Standard regimens consisting of anthracycline, cyclophosphamide, and taxane combinations are recommended [11]. However, limited information is available regarding taxane use in PABC.

For HER-2 positive breast cancers, trastuzumab and tamoxifen are useful. Trastuzumab is associated with oligohydramnios and anhydramnios, so it is generally recommended to be delayed after fetal delivery [11]. However, a recent study found 20% of cases associated with renal toxicity during the first trimester compared to over 70% of cases after the first trimester [12]. Close monitoring can be considered if trastuzumab is required during the first trimester. Endocrine therapy such as tamoxifen, are associated with a high frequency of congenital malformations so it is recommended to avoid pregnancy up to three months after its use [13,14].

Recent studies have reconsidered taxane use in PABC. Docetaxel shares the same binding site as paclitaxel but with a 1.9 times greater affinity [15]. Taxanes stabilize microtubules, inhibiting cell division. Placental crossing of taxanes seems to be limited, with placental P-glycoprotein transporter reducing passage to the fetus [16]. There may be limited data due to the preference to use anthracyclines. Adding taxanes to anthracycline regimens has been found to not result in less favorable obstetric and fetal outcomes, with a 2.2% fetal malformation rate beyond the first trimester [17]. Taxanes may be added to regimens in the second and third trimesters.

### 3.2. Clinical Evidence on Taxane Use During Pregnancy

#### 3.2.1. Taxanes

The use of taxanes during pregnancy has become increasingly recognized as a viable treatment option for various cancers. Taxanes are primarily indicated for breast cancer [17,18,19], but also for ovarian [8,20] and cervical cancers [20,21]. Clinical consensus and available data strongly support the administration of taxane-based chemotherapy after the first trimester of pregnancy, typically in the second or third trimester (gestational age ≥14 weeks) [17,18]. Median gestational ages for initiating taxane therapy were reported between 17 and 28 weeks [8,17,18,19]. This time window is crucial to minimize potential teratogenic risks associated with early embryonic development.

#### 3.2.2. Paclitaxel

Paclitaxel is an extensively used taxane in the treatment of breast and other cancers [17], often in combination with other chemotherapy drugs or targeted monoclonal antibodies. While paclitaxel was not initially recommended for breast carcinoma during pregnancy [22], more recent evidence supports comparable outcomes and risks for the use of paclitaxel relative to other immunotherapies. A case series of 18 pregnant patients receiving weekly paclitaxel by Girardelli et al. demonstrated only minor complications [18]. Reported obstetric complications during paclitaxel treatment include intrauterine growth restriction [17], preterm premature rupture of membranes (PPROM) [17], low birth weight and small for gestational age (SGA) [18], and gestational diabetes mellitus [17]. However, the incidence and nature of these complications vary across cohorts described in literature. Sella et al. demonstrated that a composite maternal-fetal risk score derived from the complications was not significantly associated with taxane use during pregnancy [17]. Maternal complications of paclitaxel treatment include hypersensitivity reactions, neutropenia, thrombocytopenia, and nausea [17]. Chemotherapy-induced amenorrhea is another potential consequence of chemotherapy. Recovery was higher in younger age (<40 years) and the taxane-based chemotherapy compared to a cyclophosphamide-methotrexate-5-fluorouracil regimen [23]. The risk of amenorrhea is greater in women treated with taxanes followed by doxorubicin and cyclophosphamide compared to doxorubicin and cyclophosphamide only [24]. The risk of congenital complications is another factor for the consideration of paclitaxel use during pregnancy. In a cohort of 103 patients with breast cancer, congenital malformations were reported in only 2.2% of neonates exposed to taxanes [19].

#### 3.2.3. Docetaxel

While less described in the literature than paclitaxel, case reports featuring treatment with docetaxel suggest it is reasonably well tolerated during pregnancy. Docetaxel proved useful in the treatment of anthracycline-resistant metastatic breast cancer when it was used in pregnancy for the first time [25]. In that report, the infant was delivered with a normal birthweight and Apgar score. Potluri et al. described two patients who received docetaxel with doxorubicin/cyclophosphamide with no observed, long-term fetal impacts [26]. In another case study, treatment with docetaxel following two trimester exposure to tamoxifen and trastuzumab led to no subsequent fetal malformations or obstetric complications [27].

Concomitant treatment with docetaxel and the HER-2 binding monoclonal antibodies trastuzumab or pertuzumab in patients with breast cancer showed evidence of possible oligohydramnios and anhydramnios risk [28,29]. However, this is mostly likely attributed to the monoclonal antibody agents, since Gottschalk et al. found that combination therapy of trastuzumab with carboplatin/docetaxel also resulted in fetal renal insufficiency and anhydramnios. The adverse effect of trastuzumab on the fetal renal function has been well documented in literature [13] and was shown to be reversible upon termination of treatment, confirming the current recommendation that use of trastuzumab in pregnancy should be avoided [30].

#### 3.2.4. Anthracyclines and Other Combined Regimens

Anthracyclines are another class of cytotoxic chemotherapy drugs which have seen wide use in the treatment of breast cancer. The sequential administration of anthracyclines followed by taxanes is a common treatment strategy, and this approach has been extended to pregnant patients. This regimen is primarily indicated for cases requiring more aggressive treatment or when there is a higher risk of recurrence [31]. Studies evaluating this combined approach have generally reported reassuring outcomes. In a cohort of 36 patients with PABC receiving doxorubicin/cyclophosphamide, low rates of maternal and fetal complications were observed, although over half of deliveries occurred pre-term [32]. Similarly, treatment with anthracyclines in two other cases of PABC, delivery was induced before the 37th week of gestation. One infant presented low birth weight, and the other infant had no complications observed [33].

#### 3.2.5. Anthracyclines vs. Taxanes

Data from a 20-year international cohort study revealed a relationship between platinum-based chemotherapy and small for gestational age, and between taxane chemotherapy and NICU admission. The NICU admission seemed to depend on cancer type, with gastrointestinal cancers having highest risk and thyroid cancers having lowest risk when compared with breast cancer [34]. PPROM or preterm contractions, small for gestational age and NICU admission had higher odds ratio with taxanes compared to anthracyclines [34]. However, it is plausible that the NICU admissions may be due to confounding factors, namely prematurity. A retrospective review on the occurrence of subsequent pregnancies in women after chemotherapy for breast cancer revealed that anthracyclines were associated with a greater probability of pregnancy compared with a taxane-containing regimen [35].

The current evidence base for chemotherapy use in pregnancy, particularly for taxanes and platinum agents, is growing but still relies heavily on case reports and smaller cohort studies. Future research should prioritize expanding data through continued prospective data collection via registries and multicenter studies to increase sample sizes and provide more robust evidence on the safety and efficacy of various chemotherapy agents and combinations.

### 3.3. Maternal and Fetal Outcomes

Current evidence from systematic reviews, registry data, and large international cohorts indicates that administering taxane-based chemotherapy after the first trimester does not alter maternal or fetal complication rates compared to conventional anthracycline-based chemotherapy [17,22,36]. In an international cohort study of 103 patients with breast cancer receiving taxanes primarily during the third trimester, [17] reported a congenital anomaly rate of 2.2% amongst 93 live-born infants, with no consistent malformation pattern identified. These findings are consistent with a larger systematic review conducted by [36], which reported a 5% malformation rate, a figure comparable to both the general population and anthracycline-only cohorts as documented in an earlier study by [8]. Similarly, the prevalence of intrauterine growth restriction (IUGR), following taxane exposure (8.5%), was aligned with previously reported rates for anthracycline-treated pregnancies [8]. Collectively, these data suggest that taxanes are not associated with teratogenic effects when administered after completion of organogenesis in the first trimester.

The most frequently observed adverse fetal outcomes following taxane exposure included preterm birth and related neonatal complications. Both [17,36] reported high rates of preterm delivery in taxane-exposed pregnancies, at 43% and 54.5% respectively. These rates are comparable to the 47% rate observed by [22] in patients receiving anthracycline-based regimens. Notably, the most preterm deliveries were iatrogenic and medically planned for oncologic or obstetric management rather than spontaneous preterm labor. The reported neonatal complications most commonly included acute respiratory distress syndrome, hyperbilirubinemia, and hypoglycemia [17,36]. Approximately 16% of neonates required admission to a neonatal intensive care unit (NICU), a rate comparable to that observed in anthracycline-treated populations [17]. Conversely, a large prospective cohort study carried out by [34] involving 1,170 pregnant cancer patients found higher NICU admission rates and increased incidence of small-for-gestational age (SGA) neonates amongst those exposed to taxane-containing regimens as compared with anthracycline-only regimens. However, this effect appears largely attributable to early delivery timing rather than a direct effect of drug toxicity.

Long-term follow-up data on the effects of in-utero taxane exposure beyond the neonatal period remain limited and based on small cohorts. Ref. [36] reported a median follow-up of 16 months, during which 13 of 86 children exhibited complications such as speech delays and, in rare cases, secondary malignancies such as acute myeloid leukemia. Ref. [17] provided longer-term follow-up for 28 children at a median period of 42 months, reporting that 85.7% were healthy, a rate exceeding that of children exposed to other chemotherapy agents (57%). Amongst the few children with abnormalities, three of four were born preterm, further suggesting that the complications may be linked to prematurity rather than taxane exposure itself. Nevertheless, larger prospective studies with extended follow-up are necessary to fully characterize long-term safety.

Maternal outcomes associated with taxane use during pregnancy are favorable when therapy is administered after the first trimester as part of standard protocol. Survival rates for pregnant women treated with taxane-containing regimens are comparable to nonpregnant counterparts when treatment is delivered according to established guidelines [37]. When dosed correctly based on body surface area, anthracycline-taxane regimens maintain efficacy without diminishing oncologic outcomes [38]. Furthermore, current consensus guidelines recommend against delaying chemotherapy until postpartum, as these delays may increase relapse risk [39].

The maternal toxicity profile of taxane-based chemotherapy in pregnancy is consistent with that observed in nonpregnant patients. Reported adverse effects include neuropathy, hypersensitivity reactions, neutropenia, anemia, and fatigue, with grade 3–4 toxicities occurring infrequently [17]. Rates of hematologic toxicities, such as anemia, thrombocytopenia, and neutropenia in pregnant patients mirror those seen in the general population [36]. Common chemotherapy-related symptoms such as nausea and fatigue remain manageable and are not exacerbated by pregnancy [17]. Potential biases in registry data include selection bias, confounding by indication, small sample sizes, heterogeneity of regimens, and incomplete follow-up data. Selection of bias occurs as most published cohorts and registries focus on women who were able to receive chemotherapy in first trimester and do not often include those with poor performance status or advanced disease, which can underestimate adverse outcomes. Confounding by indication occurs as taxanes are frequently administered with other cytotoxic agents, thus making it difficult to attribute outcomes or toxicites solely to taxanes [38]. Small sample sizes and the rarity of cancer in pregnancy may undermine the statistical power of the studies [8,17,29]. Heterogeneity of regimens refers to variability in taxane time, dosing schedules, and usage of various growth factors may complicate direct comparison or generalizability. Finally, incomplete follow-up and limited long-term data restricts the extensive application of the data. Although these biases need to be considered, the current data continues to collectively support the safety of taxane-based chemotherapy during pregnancy when clinically indicated and administered in accordance to current recommendations.

## 4. Conclusions

The available evidence demonstrates that taxane-based chemotherapy administered after the first trimester provides a safe and effective treatment option for patients with pregnancy-associated breast cancer. Maternal survival outcomes are comparable to those of nonpregnant patients when treatment follows standard oncologic protocols, without increasing fetal or neonatal complications. The rates of congenital anomalies, growth restriction, and neonatal health issues in taxane-exposed infants are similar to those observed with anthracycline-based protocols and within the general population, suggesting that drug toxicity is not a major contributor to fetal risk.

Postpartum treatment delay is not necessary, as timely therapy during pregnancy ensures optimal maternal outcomes without compromising fetal safety. Future research should focus on prospective multicenter studies with long-term follow-up to better characterize developmental outcomes in exposed offspring. Clinicians are encouraged to follow evidence-based guidelines such as those from the NCCN and ESMO, which support the use of anthracycline–taxane regimens in the second and third trimesters for appropriate candidates.

## Data Availability

This article is based on previously published studies that are publicly available and properly cited within the manuscript. No new datasets were generated or analyzed during the current study.
